# Genetic diversities of cytochrome B in Xinjiang Uyghur unveiled its origin and migration history

**DOI:** 10.1186/1471-2156-14-100

**Published:** 2013-10-09

**Authors:** Abdurahman Ablimit, Wenbei Qin, Wenjuan Shan, Weiwei Wu, Fengjun Ling, Kaitelynn H Ling, Changjie Zhao, Fuchun Zhang, Zhenghai Ma, Xiufen Zheng

**Affiliations:** 1 Life science and technology, Xinjiang University, Xinjiang Key Laboratory of Biological Resources and Genetic Engineering, Urumqi 830046, China; 2 DNA Laboratory, Institute of Forensic Science of Zhejiang, Hangzhong, China; 3 Beijing Entry-Exit Inspection and Quarantine Bureau, Beijing, China; 4 Department of Pathology, University of Western Ontario, London, Ontario N6A 5A5, Canada; 5 Central University of Finance & Economics, Beijing, China; 6 Lawson Health Research Institute, London, Canada; 7 University of Western Ontario, Lawson Health Research Institute, London, Canada

**Keywords:** Mitochondrial DNA, Cytochrome B, Uyghur, Genetic diversity

## Abstract

**Background:**

Uyghurs are one of the many populations of Central Eurasia that is considered to be genetically related to Eastern and Western Eurasian populations. However, there are some different opinions on the relative importance of the degree of Eastern and Western Eurasian genetic influence. In addition, the genetic diversity of the Uyghur in different geographic locations has not been clearly studied.

**Results:**

In this study, we are the first to report on the DNA polymorphism of cytochrome B in the Uyghur population located in Xinjiang in northwest China. We observed a total of 102 mutant sites in the 240 samples that were studied. The average number of mutated nucleotides in the samples was 5.126. A total of 93 different haplotypes were observed. The gene diversity and discrimination power were 0.9480 and 0.9440, respectively. There were founder and bottleneck haplotypes observed in Xinjiang Uyghurs. Xinjiang Uyghurs are more genetically related to Chinese population in genetics than to Caucasians. Moreover, there was genetic diversity between Uyghurs from the southern and northern regions. There was significance in genetic distance between the southern Xinjiang Uyghurs and Chinese population, but not between the northern Xinjiang Uyghurs and Chinese. The European vs. East Asian contribution to the ten regional Uyghur groups varies among the groups and the European contribution to the Uyghur increases from north to south geographically.

**Conclusion:**

This study is the first report on DNA polymorphisms of cytochrome B in the Uyghur population. The study also further confirms that there are significant genetic differences among the Uyghurs in different geographical locations.

## Background

Uyghur is one of the populations living in central Asian where people have undergone unceasing migration and interacted with other populations in prehistoric and historic times. As a consequence, Uyghurs share anthropometric and genetic traits with Eastern and Western Eurasian. They are genetically considered to be an admixture of Eastern and Western Eurasian populations, demonstrated by archaeological, anthropologic and genetic studies [1-6]. The Uyghur population has been used as a good human population sample for studying the human migration and gene inflow/drafting [3,7]. However, the origin of the Uyghur is under discussion. Modern Uyghurs live primarily in the Xinjiang Uyghur Autonomous Region in China. The majority of the Xinjiang Uyghur live in the south of Xinjiang (south of Tian Shan), with minor populations spreading to the north. Due to environmental, war and political reasons, Uyghurs have undergone unceasing migration (from north to southwest) and interacted with other populations in prehistoric and historic times. This might result in genetically differential characteristics of modern Uyghurs living in different regions. However, most genetic studies of the Uyghur have focused on genetic difference between them and other ethnic groups, and utilized relatively small numbers of sample from very limited geographic locations. The differences in genetic characters between different regional Uyghur populations remain largely unclear. Furthermore, previous studies mainly investigated and compared the genetic diversity of mtDNA control region sequences, while DNA polymorphism of mitochondrial Cytochrome B (CYTB) in the Uyghur population in Xinjiang has not been reported.

CYTB is located in the coding region of mtDNA (positions 14576–16047), spanning the 1140 bp fragment. CYTB has undergone changes during evolution resulting in the occurrence of multiple single nucleotide polymorphic sites [8,9]. Compared with the control region of mtDNA, CYTB is relatively conservative and has fewer mutations [8,10]. CYTB is widely used in taxonomic research to determine phylogenetic relationships between organisms due to its sequence variability [11-13]. It is also considered to be most useful in determining relationships within families and genera. CYTB sequence information drawn from populations reflects their matrilineal ethnohistory and can deduce DNA sample's population based on population-specific nucleotide mutations existing in the CYTB gene [9,14]. Therefore, we hypothesized that the genetic diversity of Uyghur living in different regions of Xinjiang can be identified by the analysis of CYTB sequence.

In this study, we for the first time report on the gene diversity of CYTB in the Uyghur of Xinjiang by completely sequencing their CYTB gene. We demonstrate that there was a genetic variance of CYTB occurring in the different regional Uyghurs and CYTB is a very useful genetic marker for the study of genetic differentiation of Uyghurs in Xinjiang. In particular, we showed the founder/ bottleneck event in the Xinjiang Uyghur.

## Results

### High gene diversity of cytochrome B in the Uyghur population

1190 bp PCR fragments were amplified in all studied samples and 1140 bp of DNA fragment encoding CYTB (positions 14747–15886) were sequenced. Compared with the standard reference sequence (rCRS), a total of 102 mutant sites were observed in the studied 240 samples (Additional file 1: Table S1). The number of mutation sites in the samples varied from 0 to 10 with an average of 5.126. There were no deletions and/or insertions observed.

**Figure 1:**
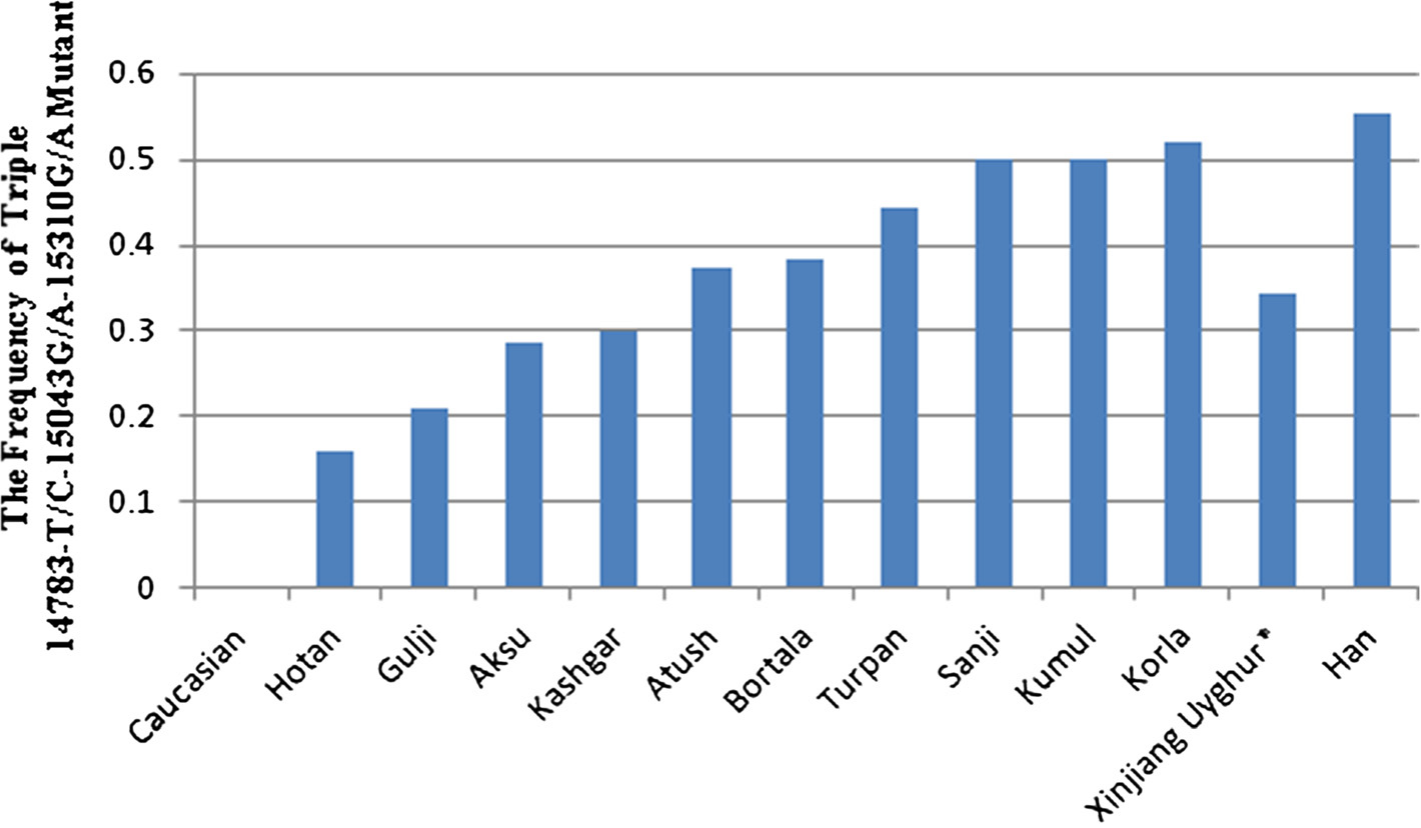
**The frequency of triple 14783 T/C-15043G/A-15301G/A mutant.** DNA was isolated from 240 samples collected from ten different locations of Xinjiang and sequenced. DNA sequences were compared with the standard reference sequence using MEGA5.0, as described in the methods. The frequency of nucleotide mutation was calculated.

Table 1 lists the most common and group-unique mutation positions. Position 15326 A –G transition occurred in almost all samples except 2 samples (one from Sanji and another one from Bortala). The second highest mutation position was nt 14766 C-T with an average frequency of 82.92% observed in 240 studied Uyghur individuals. More than 90% of Uyghur from Korla, Kumul, Sanji and Turpan had a 14766 C-T mutation, while this mutation occurred less than 80% in the individuals from Aksu (77.14%), Gulja (73. 68%), Hotan (78.95%), and Kashgar (79.10%). The third dominant mutation positions were 14783 T-C (36.25%), 15043 G-A (36.25%) and 15301 G-A (35.42%). Position 14783 T/C, 15043G/A, and 15301 G/A mutations simultaneously took place with an average frequency of 34.17% in the overall Uyghurs. The frequency of the triple 14783 T/C-15043G/A-15301G/A mutant varied from population to population, from region to region (Figure 1). In general, the frequency of the triple 14783 T/C-15043G/A-15301G/A mutant among the ten regional subpopulations decreased in the order of Kumul, Sanji, Turpan, Bortala, Atush, Kashgar, Aksu and Hotan, in line with their geographic location from north to south. The lowest frequency of this triple mutant (15.79%) was observed in the group from Hotan which is located in the south end of Xinjiang, adjacent to Tibet, India and Pakistan. The north end regions, geographically closer to Mongolia, had similar frequencies of the triple 14783 T/C-15043G/A-15301G/A mutant to the Mongolian people(our unpublished data).

**Table 1:** The frequency of population-specific polymorphic mutant nucleotides

**rCRS**	**Nucleotide**	**Amino acid**	**Aksu**	**Atush**	**Bortala**	**Gulja**	**Hotan**	**Kashgar**	**Korla**	**Kumul**	**Sanji**	**Turpan**	**Han**	**Caucasian**
**position**	**change**	**change**	**(35)**	**(24)**	**(13)**	**(19)**	**(19)**	**(67)**	**(25)**	**(10)**	**(10)**	**(18)**	**(56)**	**(24)**
14766	C → T	T7I	0.771	0.875	0.846	0.734	0.789	0.7910	0.920	0.900	0.900	0.944	1.00	0.833
14783	T → C	L13L	0.285	0.458	0.384	0.263	0.158	0.313	0.520	0.500	0.500	0.500	0.554	
14798	T → C	F15L	0.029				0.052	0.015					0	0.208
14857	T → C	L37L										0.111		
14927	A → G	T60A			0.154							0.055	0.0179	
15043	G → A	G99G	0.314	0.375	0.384	0.210	0.210	0.313	0.520	0.600	0.500	0.500	0.554	0.083
15301	G → A	L185L	0.285	0.458	0.384	0.263	0.158	0.298	0.520	0.500	0.500	0.444	0.571	
15326	A → G	A193A	1.000	1.000	0.923	1.000	1.000	1.000	1.000	1.000	0.900	1.000	1.000	1.000
15440	T → C	L231L			0.154				0.040			0.056	0.0179	
15746	A → G	I333V								0.300				0.0418

Other populations’ data are cited from [14].

Studying mutations among the regions, we observed the presence of regional group specific mutations in the CYTB sequence (Table 1). The mutations 14927 A/G and 15440 T/C were only observed in the Bortala with a frequency of 15.38%, 15746 A/G only in Kumul (30.00%), and 14857 T/C only in Turpan (11.11%). Only three samples of the total 240 Uyghurs showed a nucleotide change at position 14798 which occurs more frequently in Caucasians with a frequency of 0.2083, but not in other Asian groups [14].

The genetic diversities in the regional population were calculated (Table 2). The gene diversities varied among groups from 0.8800 in Korla to 0.97449 in Bortala, while nucleotide diversities varied from 0.00269 (in Sanji) to 0.007863 (in Korla), respectively. The discrimination power (DP) was higher than 0.8200, even up to 0.9454 (in Kashgar). The overall genetic diversity and power of discrimination were 0.9480 and 0.9440, respectively, suggesting that MYTC B is one of the highly polymorphic markers useful for maternal lineage identification.

**Table 2:** The gene diversity, nucleotide diversity, and discrimination power noted in these populations

**Population**	**Gene diversity**	**Nucleotide diversity**	**Discrimination power**
Aksu	0.9546 ± 0.024	0.003450 ± 0.0019	0.9273
Atush	0.9529 ± 0.026	0.003582 ± 0.0020	0.9132
Bortala	0.9744 ± 0.038	0.004026 ± 0.0023	0.8994
Gulja	0.9591 ± 0.030	0.003052 ± 0.0018	0.9086
Hotan	0.9708 ± 0.027	0.003098 ± 0.0018	0.9197
Kashgar	0.9590 ± 0.014	0.003300 ± 0.0018	0.9454
Korla	0.8800 ± 0.051	0.007863 ± 0.0041	0.8448
Kumul	0.9333 ± 0.077	0.003957 ± 0.0024	0.8400
Sanji	0.9111 ± 0.077	0.002690 ± 0.0017	0.8200
Turpan	0.9085 ± 0.051	0.003125 ± 0.0018	0.8580
Chinese	0.8864 ± 0.034	0.0028 ± 0.0016	0.8705
Caucasian	0.9565 ± 0.022	0.0033 ± 0.0019	0.9167

Other populations’ data are cited from [14].

### Founder / bottleneck haplotypes of CYTB in the Uyghur

**Figure 2:**
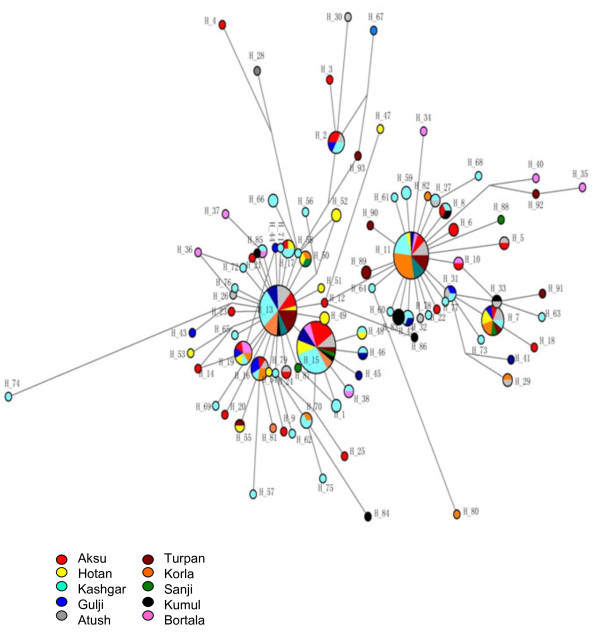
**Median-joining network.** A Median-joining network was constructed using NETWORK 4.6.1.0 software with haplotypes of CYTB from the Uyghur.

There were 93 different haplotypes observed in the 240 Uyghur samples (Additional file 2: Table S2). Due to absence of a standard for assignment of haplotypes, we called each different sequence as an individual haplotype generated by DnaSP5.0. The frequencies of different haplotypes varied between different regions (Additional file 2: Table S2). The standard reference sequence was observed only in one sample. Haplotype 15 (15326G, in which only one nucleotide mutation positioning 15326 existed) was the most dominant haplotype with a frequency of 13.75% (33/240) in the study. The second dominate haplotype was haplotype 13 (14766 T, 15326G) observed in 31 individuals out of 240 (12.92%), followed by haplotype 11 (14766 T, 14783C, 15043A, 15301A, 15326G) with a frequency of 11.25% which is the most abundant haplotype in Chinese [14]. Haplotype 7 (14766 T, 14783C, 15043A, 15204C, 15301A, 15326G, 15487 T) was observed in 11 people. Haplotype 16 (14766 T, 15326G, 15884C), as well as Haplotype 19 (14766 T, 15326G, 15693C) existed in seven individuals. Haplotype 2 (14766 T, 14905A, 15326G, 15452A, 15607G) was shared by 6 people, and haplotype 17 (14766 T, 15326G, 15535 T) shared in four people. Six sequences appeared three times (H8, H31, H42, H50, H83, and H70), and 17 sequences were observed twice. The other 62 sequences or haplotypes were only observed once. The network of haplotypes showed the existence of two major clusters (Figure 2): cluster 1 (left side of network) consisting of two-thirds of total haplotypes including H13 and H15, and cluster 2 (right side) consisting of one-third of total haplotypes including H11. The network showed that there are founder/ bottleneck haplotypes existing in the Xinjiang Uyghur which are also observed in the Mongolians (our unpublished data).

Grouping samples into different geographic locations; there were 24 haplotypes observed in 35 Aksu individuals, 16 in 24 Atush, 11 in 13 Bortala, 14 in 19 Gulja, 15 in 19 Hotan, 41 in 67 Kashgar, 13 in 25 Korla, 8 in10 Kumul, 7 in 10 Sanji, and 11 in 18 Turpan individuals. Haplotype 15 (15326G) was predominantly found in the Aksu with a frequency of 20% and in the Kashgar with a frequency of 14.93%. Haplotype 13 (14766 T, 15326G) existed most frequently in the Turpan (27.78%), Atush (16.67%) and Korla (16%), but was not in the Bortala nor Kumul. Haplotype 11 (14766 T, 14783C, 15043A, 15301A, 15326G) appeared frequently in the Korla (32%), Sanji (30%), Turpan (16.67%), and Atush (12.5%). Haplotype 83 (14766 T, 14783C, 15043A, 15301A, 15326G, 15746G) was only observed in the Kumul group with a frequency of 30%, but not in other groups thereby showing region-specificity. Haplotype 89 (14766 T, 14783C, 14857C, 15043A, 15301A, 15326G) was observed only in the Turpan with a frequency of 11.11%, while haplotype 59 (14766 T, 14783C, 15043A, 15301A, 15326G, 15673A) and haplotype 66 (14766 T, 15314A, 15326G, 15452A) were only found in the Kashgar group with a frequency of 2.99%, respectively.

### Different regional Uyghur represent different genetic features

**Figure 3:**
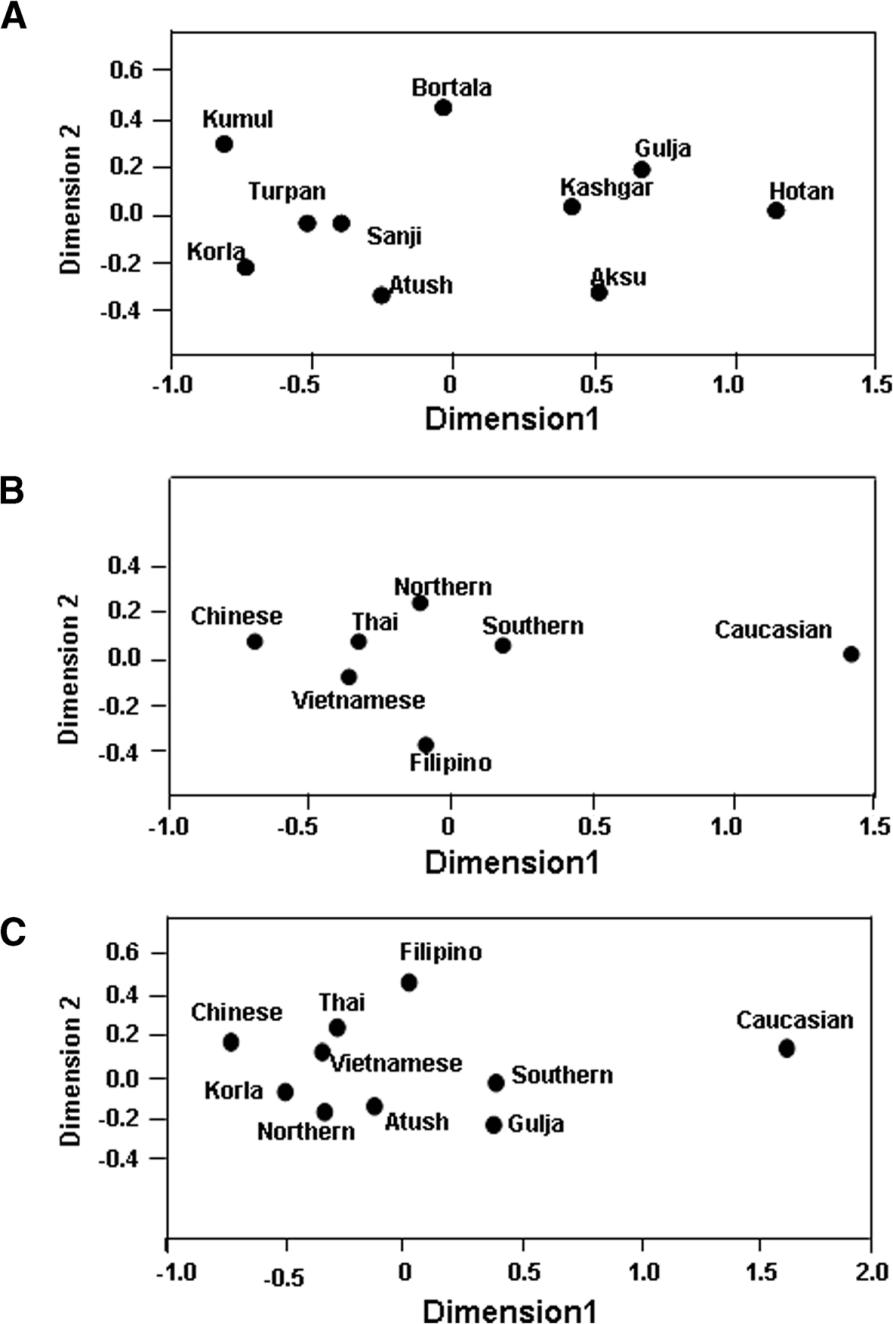
**Multiple dimension scale plot by SPSS. (A)** MDS plot of ten different regions' Uyghur people. **(B)** MDS plot of geographic-based southern and northern Uyghur population. **(C)** MDS plot of regrouped populations. Other populations were cited from [14].

In order to investigate the genetic diversity of CYTB in ten regional Xinjiang Uyghurs, MDS analysis was first conducted within ten regional Xinjiang Uyghurs and groups were made based on their geography. As shown in Figure 3A, ten regions were separated from each other by dimension 1 and some subpopulations were gathered closer than others. Kumul, Turpan, Sanji, Atush and Korla were located closely at the left side in the MDS plot forming cluster 1, while Kashgar, Aksu, Hotan and Gulja sit on the right side and formed cluster 2. Bortala was almost in the middle in the MDS plot. However, in dimension 2, Bortala was closely distributed to Kumul (a northern Xinjiang place), but still be separated from cluster 2 consisting of most of southern Xinjiang regions.

Tianshan and the ancient Silk road are a geographic dividing line separating Xinjiang into two parts: Southern Xinjiang (Nanjiang) and Northern Xinjiang (Beijiang). Nanjiang consists of Kashgar, Aksu, Hotan, Atush and Korla, while Kumul, Turpan, Sanji, Gulja and Bortala belong to Beijiang. Accordingly we divided ten regional Xinjiang Uyghurs into two subgroups: Southern Uyghur (groups living in Nanjiang) and Northern Uyghur groups (living in Beijiang). We then performed MDS analysis with the Southern and Northern Uyghur and other populations [14-18] in order to reveal the genetic relationships among these populations. Figure 3B shows that both Northern and Southern Uyghurs and other 4 Asian populations (Thai, Vietnamese, Filipino and Chinese) were distributed closely to the left in MDS plot and formed a cluster, while Caucasians were isolated far away from them and the cluster, suggesting that both Southern and Northern Uyghur are closer to Asian in genetics than to Caucasian. In addition, the Northern Uyghur was separated from the Southern Uyghur. In comparison with the Southern Uyghur, the Northern Uyghur were more closely located to the Chinese.

Furthermore, we noticed that Gulja Uyghurs who geographically reside in northern Xinjiang appeared closely related to the southern subgroups (Hotan, Kashgar and Aksu) in the MDS plot, while geographic southern Uyghur subgroups Korla and Atush were close to the northern subgroups (Kumul, Sanji and Turpan) as shown in Figure 3A. Therefore we regrouped Uyghur samples by subtracting Gulja from northern group, Korla and Atush from the southern group, and conducted the third MDS analysis using new designed groups (Figure 3C). This regrouped MDS plot displayed that the northern group (Turpan, Kumul, Sanji and Bortala) was separated further away from the southern group (Hotan, Aksu and Kashgar). Korla was distributed between Chinese and the new northern group with similarly close distance to both of them. The Gulja and the new southern group formed a cluster, clearly separated from the northern group and Caucasian. In contrast, Atush was distributed relatively more closely to northern Uyghur.

### The patterns of haplotypes of CYTB reflected migration history and origin of Uyghur

**Figure 4:**
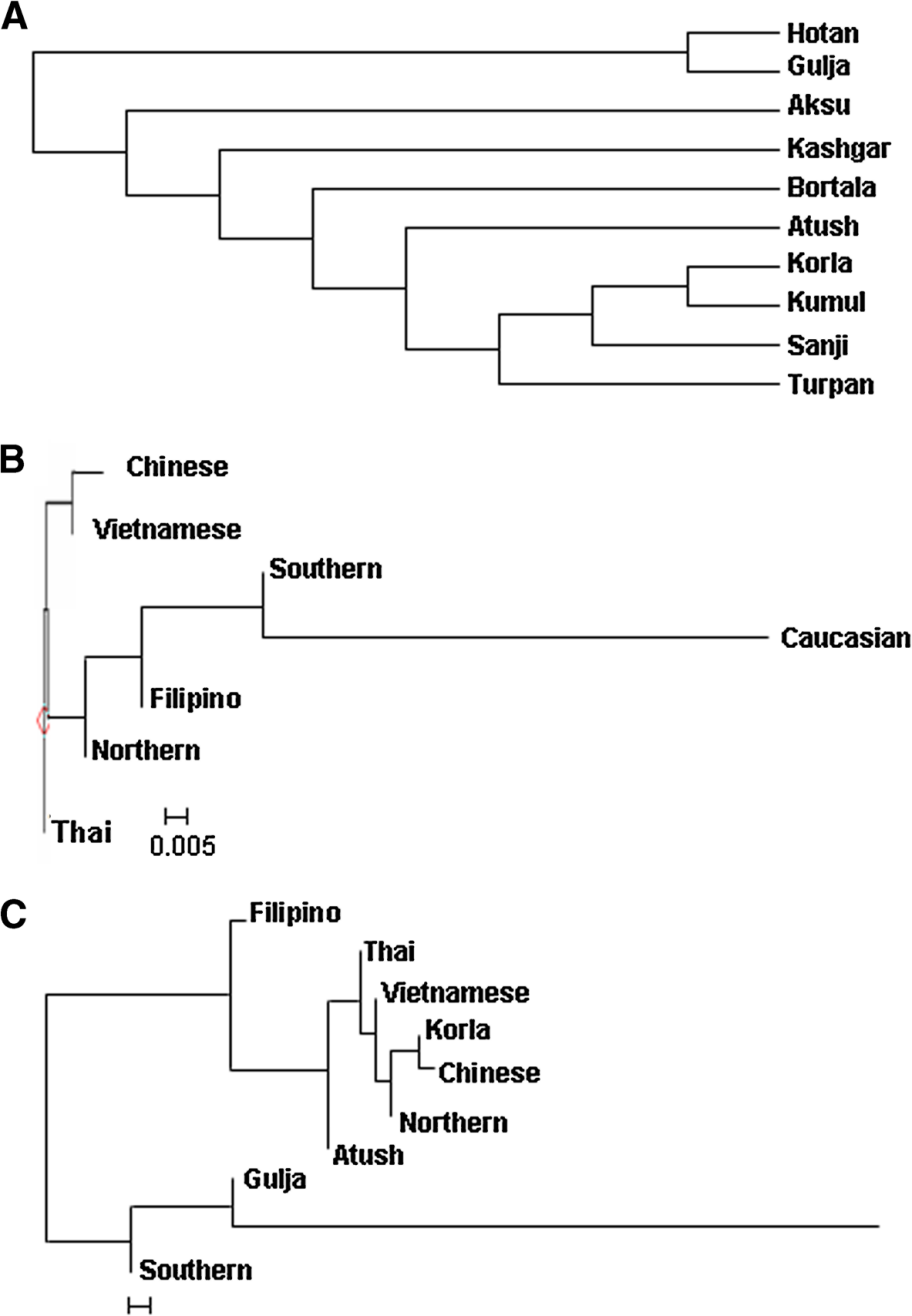
**Unrooted Neighbor-joining tree.** Using genetic distance in Table 3**(A)**, Table 4**(B)** and Table 5**(C)** an unrooted Neighbor-joint trees were constructed using Phylips 3.6.9 software. Other populations were cited from [14].

To further explore the genetic linkage, the genetic distances between each of the regional groups were determined (Table 3). Genetic distances between regions shown in Table 3 nicely reflected their geographic distance. There were significant differences in genetic distance between south region Hotan and north regions Kumul and Turpan. Interestingly, there were significant differences between the south region Korla and Hotan and between Korla and Kashgar. An unrooted NJ tree was constructed based on the ten regional groups’ F_ST_ distances as seen in Table 3 (Figure 4A). The tree showed that there were three branches: Hotan and Gulja formed one branch; Turpan, Sanji, Kumul and Korla formed the second branch, Aksu, Kashgar, Bortala and Atush formed the third Branch. The tree also showed that there were genetic diversities within branches with the exception of the first cluster.

**Table 3:** Genetic distances between ethnic groups

	**Aksu**	**Atush**	**Bortala**	**Gulja**	**Hotan**	**Kashgar**	**Korla**	**Kumul**	**Sanji**	**Turpan**
Aksu	0.00000									
Atush	0.00211	0.00000								
Bortala	−0.00080	0.00514	0.00000							
Gulja	−0.02210	0.00407	−0.00115	0.00000						
Hotan	−0.01177	0.04014	0.02028	−0.02148	0.00000					
Kashgar	−0.01114	0.00789	0.00089	−0.01570	0.00161	0.00000				
Korla	0.03360	−0.01535	0.00972	0.03474	0.07861*	0.02696*	0.00000			
Kumul	0.03582	−0.00575	0.00173	0.05026	0.08220*	0.03259	−0.01094	0.00000		
Sanji	0.00002	−0.04272	−0.02413	0.00965	0.04600	−0.00418	−0.05257	−0.03620	0.00000	
Turpan	0.01837	−0.02520	−0.00876	0.02662	0.05889*	0.01663	−0.02840	−0.01697	−0.05287	0.00000

*p-value < 0.05.

With the same strategies in MDS analysis, we regrouped samples and calculated genetic distance (Tables 4 and 5) and constructed phylogenetic trees (Figure 4B and 4C) using new assigned groups. Table 5 showed the results of genetic distance generated by regrouping the Uyghur into two major groups--Southern and Northern Uyghur. Figure 4B and 4C are NJ trees constructed with the genetic distances from Tables 4 and 5. We found that both the southern and northern groups showed shorter genetic distance from Chinese than from Caucasian. There were significance in genetic distance between the southern Uyghur and Caucasian, between the southern Uyghur and Chinese, and between the northern Uyghur and Caucasian, while there was no significance between the northern Uyghur and Chinese. The NJ tree (Figure 4B) intuitively showed the genetic relationship between these populations, supporting Table 4 results. Table 5 further confirmed the genetic relationship between the southern, northern, Chinese and Caucasian. More importantly, Table 5 and Figure 4C demonstrated that geographic southern cities Atush and Korla showed gene similarity to the northern Uyghur, although northern city Gulja is genetically similar to the Southern Uyghur, but not to the northern Uyghur. The Korla Uyghur was completely different to the southern Uyghur (Hotan, Aksu and Kashgar).

**Table 4:** Genetic distances between ethnic groups

	**Southern**^**a**^	**Northern**^**b**^	**Thai**	**Vietname**	**Filipino**	**Caucasian**	**Chinese**
Southern^a^	0.00000						
Northern^a^	0.00086	0.0000					
Vietname	0.00651	−0.00862	0.0000				
Thai	0.01162	−0.00412	−0.01779	0.0000			
Filipino	0.02584	0.01948*	0.00471	0.00535	0.0000		
Caucasian	0.09864*	0.13940*	0.17506*	0.16400	0.12715*	0.0000	
Chinese	0.04366*	0.01697	−0.00875	−0.00216	0.03184*	0.23711*	0.00000

^a^: Southern Uyghur including Aksu, Kashgar, Korla, Hotan, Atush; ^b^:Northern Uyghur including Kumul, Turpan, Gulja, Sanji, Bortala, based on geographic definition. Other populations’ data are cited from [14]. *p-value < 0.05.

**Table 5:** Fst based on Tajia Nei

	**Southern**^**a**^	**Korla**	**Atush**	**Gulja**	**Northern**^**b**^	**Vietnamesec**^**c**^	**Thai**^**c**^	**Filipino**^**c**^	**Caucasian**^**c**^	**Chinese**^**c**^
Southern	0.0000									
Korla	0.03858*	0.0000								
Atush	0.01483	−0.01542	0.0000							
Gulja	−0.01681	0.03568	0.00410	0.0000						
Northern	0.02872*	−0.01415	−0.00680	0.02597	0.0000					
Vietnamese	0.02297*	−0.01334	−0.00698	0.03586	−0.01324	0.0000				
Thai	0.02888*	−0.01165	−0.00783	0.03315	−0.00801	−0.01779	0.0000			
Filipino	0.03147*	0.02730	0.02617	0.03233	0.02005	0.00471	0.00535	0.0000		
Caucasian	0.07257*	0.19982*	0.142818	0.06682*	0.16749*	0.17506*	0.1640*	0.12715*	0.0000	
Chinese	0.06878*	80.00875	0.01067	0.08009*	0.00258	−0.00875	80.00216	0.03184	0.23711*	0.0000

^a^: Southern Uyghur: including Aksu, Kashgar, and Hotan; ^b^: Northern Uyghur: including Kumul, Turpan, Sanji and Bortala, based on geographic definition. ^c^: Other populations’ data are cited from [14]. *p-value < 0.05.

## Discussion

In this study we first reported the genetic polymorphisms of cytochrome B in Uyghur population of Xinjiang. We demonstrated that cytochrome B is a very useful and informative marker for matrilineal identification and population studies, second to the mtDNA control region. There are population specific and regional population specific nucleotide positions in the cytochrome B sequence, which can help identify DNA sample's region and population information. The results of the present study conclude that: 1) there are founder/ bottle neck events in Xinjiang Uyghur; 2) the Uyghur is closer to Chinese, rather than to Caucasian in genetic distance; 3) There are genetic difference between the Southern and Northern Uyghur; 4) Gene influence from Asian (Chinese) is stronger on the Northern Uyghur, than the Southern Uyghur; and 5) CYTB is a good genetic marker for differentiation of subgroups.

Xinjiang is located in Central Asia which is an intermediate region of the Eurasian continent. There were 36 nations existing in Xinjiang during prehistorical and historical times. Some nations were defeated by others and destroyed, overrun or forced to migrate to other places. Unceasing wars propelled endless migration and coalition, leading to genetic mixture and gene drafting. Additionally, there were two silk roads extending from east to west through Xinjiang, resulting in extensive exchange of culture and marriage between different populations. Our data confirm that Uyghur is an admixture population with contributions from both Eastern and Western Eurasian ancestries, which is consistent with the results from mtDNA control region sequences [3-6]. Based on the mutation at position 14798, the CYTB sequences of the Uyghur in Xinjiang contain more inflow of East Asian than European. The proportion of European sequences varies from different geographic regions of the Uyghur (data not shown).

The Uyghurs are a Turkic ethnic group living in Eastern and Central Asia. The ancestors of the Uyghur tribe were Turkic pastoralists called Tiele in Northern China, Mongolia, and the Altay Mountains. Due to wars and environmental stress, Uyghur continued migrating from north to south. From the historic perspective, Uyghur originated from Mongolians, rather than Caucasians and inherited Mongolia genetics. The Uyghur population was gradually diluted as they migrated from north to south. Our data on the frequency of triple 14783 T/C-15043G/A-15301G/A mutant and founder/bottle neck haplotypes demonstrated that the Uyghur originated from Mongolia, migrated from north to south, supporting historic reports.

In this study, we found that nucleotide positions with most frequent mutation were 14766, 14783, 15043, and 15302, which were most commonly seen in Asians including the Chinese Han population [14]. Mutation at position 14766 occurs in more than 93% of Asian samples, but less than 0.72% of Caucasians [8,14]. The frequencies of mutation at the position 14766 in the ten different geographic Uyghur samples varied from 0.73 to 0.94. The frequencies of 14766 mutation declined from the eastern Tarim Basin to the western Tarim Basin, reflecting a clear geographic pattern. This implies that the East Asian portion of genetics was gradually diluted as people migrated from the east to the west of Xinjiang, and from north to south. Mutations occurring at position of 14783, 15043, and 15302 showed the same trend among the ten Uyghur groups as position 14766. Mutations at the above three positions were seldom observed in the Caucasian population. In contrast, mutation at position 14798 that is Caucasian specific was observed in only one Aksu sample and two Hotan samples. Taken together, we found that at the cytochrome B gene, Uyghurs generally have more imprint of East Asian genetic portion than that of Caucasian. These genetic results are in agreement with the history of Uyghur formation. The Uyghurs living in the Nanjiang are different to Beijiang, and the southern Uyghur have relatively less influence from East Asians than the northern. Our data showed that in the same ethnic group, people residing at different places have genetic differences.

In this study, we reported that Uyghur from different geographic locations in Xinjiang have differences in the percentage of European/East Asian ancestry component, distinctly by the difference between Aksu/Hotan and Kumul or Korla. There is a significant difference between the south (Hotan, Kashgar, and Aksu ) and the north of Xinjiang (Kumul, Bortala, Turpan and Sanji). East Asian ancestry dominates the Uyghur from the north, while European ancestry imprints more on the southern Uyghur. In addition, we observed that Uyghurs living in two geographically south cities Atush and Korla shared northern Uyghur genetics (Kumul, Turpan and Sanji), in contrast with the southern Uyghur, while Uyghurs from Gulja which is located in the north of Tianshan in geography presented the southern regional Uyghur genetic features. Demographic records show that the dominant ethnic group is Kyrgyz who defeated the Uyghurs in AD 840, despite the fact that Atush is located in the south of Tianshan [Wikipedia, the free encyclopedia, History of the Kyrgyzstan]. Moreover, there were intermarriages in the Uyghur living in the Atush region. This might explain why the gene pattern of cytochrome B in the Atush Uyghurs was between that of the southern and northern Uyghur group, different from the other three southern group samples. As for Gulja, there were two large migrations of the southern Uyghur from south to north Gulja in history. Over time, the migrated southern Uyghurs expanded in Gulja and became the largest minority in Gulja [Wikipedia, the free encyclopedia, History of the Uyghur people.]. As a result, the Gulja Uyghur was and still is a branch of southern Uyghur, having little relationship with the northern Uyghur, which is in line with our data that the Uyghur from Gulja shares similar genetic characters with the Southern Uyghur. Although Korla is located in the south of Tianshan belonging to Nanjiang ( southern of Xinjiang), it is also adjacent to Turpan and Sanji. Korla is located in Bayingolin Mongol Autonomous Prefecture where the current major population is Chinese Han. As early as 94 CE, the Chinese government and military started to administrate this area and forced different populations to exchange and mix. The results from CYTB genotyping are consistent with that from the control region of mtDNA (unpublished data). Our data showed that the Uyghurs from Korla in genetics are different to the south Xinjiang Uyghurs.

## Conclusions

Cytochrome B is a very useful DNA marker with high discriminate power for matrilineal identification, as well as deduction of the region and population of people. The polymorphisms of CYTB were significantly different between different geographical Uyghur (between south and north). The influences of East Asian and European genetics in Uyghur varies between different geographic locations (particularly south and north) of Uyghur.

## Methods

### Sampling

**Figure 5:**
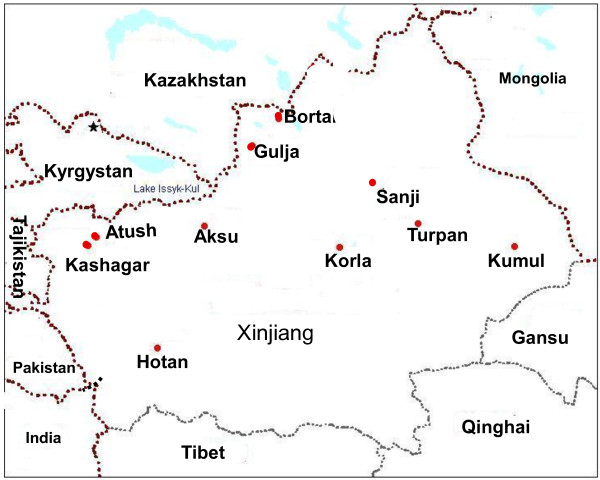
Geographic locations of the samples investigated in this study.

A total 240 healthy unrelated samples were collected from ten different Uyghur concentrated locations/communities in Xinjiang, China (Figure 5): Aksu (35), Atush (24), Bortala (13), Gulja (19), Hotan (19), Kashgar (67), Korla (25), Kumul (10), Sanji (10), and Turpan (18). We collected more samples from Kashgar due to more than 36% of Xinjiang Uyghurs live in that area. For the samples it was confirmed that there were no intermarriages between Uyghur and other ethnic groups and no migration history in the latest three generations. The study was approved by the Ethnic Study Committee of Xinjiang University. All blood samples were obtained with informed consent. DNA was extracted from blood using an EasyPure blood genomic DNA extraction kit (TransGen Biotech, Beijing, China).

### PCR and sequencing

The primers used for PCR amplification of the CYTB gene listed in Table 6 were adopted from previous studies [14] and synthesized by Shenggong (Shenggong Biotech, Shanghai, China). CYTB was amplified in a 50 μl reaction mixture containing 30 ng genomic DNA, 1 unit of Pfu DNA polymerase (Takara, Dalian, China), 200 μM dNTPs, 0.2 μM of each primers (L14724, H 15915), and reaction buffer (10 mM Tris–HCl, pH8.3, 15 mM MgCl_2_, 50 mM KCl, 0.1% gelatin (Takara). PCR was conducted in a PCR thermal cycler (Applied Biosystems, Carlsbad, CA) with the following thermal cycling conditions: pre-denaturation at 95°C for 5 min; followed by 35 cycles at 95°C 30 s; 55°C 30 s; 72°C 60 s; and final extension at 72°C for 10 min.

**Table 6:** Sequences of primers used for mitochondrial DNA amplification and sequencing

**Primer**	**Sequences(5′-3′)**
L14724^*,¶^	CGAAGCTTGATATGAAAAACCATCGTTG
H15915^*,¶^	AACTGCAGTCATCTCCGGTTTACAAGAC
L15283^¶^	TCCCACCCTCACACGATTCT
H15363^¶^	AATAGGAGGTGGAGTGCTGC

L: light strand; H: heavy strand. *: PCR; ^¶^: sequencing.

PCR products were visualized on a 1.5% agarose gel and purified using a Biomiga kit (Biomiga, Beijing, China). Sequencing reactions were conducted in a PE-9600 thermocycler (ABI Applied Biosystems, Fostor City, CA, USA), using a BigDye terminator v3.1 Cycle sequencing kit (Applied Biosystems) with the following conditions: 25 cycles of 95°C for 30 s; 50°C for 30 s; and 60°C for 4 min. The primers L14724, H15149, L15283 and H15363 were used for sequencing listed in Table 6. The DNA sequences were detected with an ABI3730 DNA sequencer (Applied Biosystems) in HuaDa Genome Centre, Beijing.

### Data analysis

Sequences were aligned using MEGA5.0 and compared with the standard reference sequence (rCRS) (http://www.megasoftware.net/mega.php). The haplotypes of CYTB were designed using DnaSP5.0. The genetic distances between Xinjiang regional Uyghur populations and other populations were determined by the analysis of molecular variance using ARLEQUIN3.5 (http://cmpg.unibe.ch/software/arlequin3). A difference with a *P*-value < 0.05 was considered statistically significant. Multidimensional scaling (MDS) plot of mtDNA was created by SPSS 19 for studying the genetic relationship between populations. Phylogenetic trees were constructed by the neighbor-joining method using a Phylip 3.69 program (http://en.bio-soft.net/tree/Phylip.html). Median networks were constructed using NETWORK 4.6.1.0 software (http://www.Fluxus-engineering.com/sharenet.htm).

## Competing interests

The authors declare that they have no competing interests.

## Authors’ contributions

AA carried out data collection and analysis. WQ carried out data analysis. WS participated in data analysis. WW, participated in data collection. FL participated in data discussion and manuscript preparation. KL participated in manuscript preparation. CZ participated in discussion of the study. FZ participated in the design of the study and coordination. ZM participated in the design of the study and sample collection. XZ participated in the design of the study, coordination, data analysis and manuscript preparation. All authors read and approved the final manuscript.

## Supplementary Material

Additional file 1: Table S1 Nucleotide mutation in the Uyghur. Click here for file

Additional file 2: Table S2 Frequency of haplotype of MTCYB. Click here for file
