# Supplementary material for: Genetic diversities of cytochrome B in Xinjiang Uyghur unveiled its origin and migration history
Source: BMC Genet. 2013 Oct 9;14:100. doi: 10.1186/1471-2156-14-100 (PMC3852047; doi:10.1186/1471-2156-14-100)
Supplement: Additional file 1: Table S1 — Nucleotide mutation in the Uyghur. [file 1471-2156-14-100-S1.doc]

|  | Aksu | Atush | Bortala | Gulja | Hotan | Kashgar | Korla | Kumul | Sanji | Turpan | Uyghur in total |
| --- | --- | --- | --- | --- | --- | --- | --- | --- | --- | --- | --- |
|  | 35 | 24 | 13 | 19 | 19 | 67 | 25 | 10 | 10 | 18 | 240 |
| 14751 |  |  | 1 |  |  |  |  |  |  | 1 | 3 |
| 14750 |  |  |  |  |  | 1 |  |  |  |  |  |
| 14753 |  | 1 |  |  |  | 1 |  |  |  |  | 3 |
| 14756 |  |  |  |  |  | 1 |  |  |  |  | 1 |
| 14763 |  |  |  | 1 |  |  |  |  |  |  | 1 |
| 14766 | 27 | 21 | 11 | 14 | 15 | 53 | 26 | 9 | 9 | 17 | 199 |
| 14767 |  |  |  |  |  | 1 |  |  |  |  | 1 |
| 14783 | 10 | 11 | 5 | 5 | 3 | 21 | 13 | 5 | 5 | 9 | 87 |
| 14788 |  |  |  |  |  | 1 |  |  |  |  | 1 |
| 14793 |  |  | 1 |  |  | 3 | 1 | 1 |  |  | 6 |
| 14794 | 1 |  |  |  |  |  |  |  |  |  | 1 |
| 14798 | 1 |  |  |  | 2 |  |  |  |  |  | 3 |
| 14801 |  |  |  |  |  |  | 1 |  |  |  | 1 |
| 14804 |  |  |  |  |  |  | 1 |  |  |  | 1 |
| 14815 |  |  | 1 |  |  |  |  |  |  |  | 2 |
| 14831 |  |  |  | 1 |  | 1 |  |  |  |  | 2 |
| 14857 |  |  |  |  |  |  |  |  |  | 2 | 2 |
| 14872 |  |  |  |  |  |  |  |  |  |  | 1 |
| 14893 |  |  |  |  |  |  |  | 1 |  |  | 1 |
| 14905 | 3 | 2 |  | 1 |  | 4 |  |  |  |  | 10 |
| 14927 |  |  | 1 |  |  |  |  |  |  | 1 | 3 |
| 14935 |  |  |  |  | 1 |  |  |  |  |  | 1 |
| 14971 |  |  |  |  |  |  |  | 1 |  |  | 1 |
| 14978 |  | 1 |  |  |  |  | 1 |  |  |  | 2 |
| 14979 |  |  |  | 1 |  | 2 |  |  |  |  | 3 |
| 15022 |  |  |  |  |  |  |  |  | 1 |  | 1 |
| 15031 |  |  |  | 1 |  |  |  |  |  |  | 1 |
| 15043 | 11 | 11 | 5 | 4 | 4 | 21 | 13 | 6 | 5 | 5 | 87 |
| 15049 | 1 |  |  |  |  |  |  |  |  |  | 1 |
| 15055 |  |  |  |  |  | 1 |  |  |  |  | 1 |
| 15061 |  |  |  |  | 1 |  |  |  |  |  | 1 |
| 15067 | 1 |  |  |  | 1 |  |  |  |  |  | 2 |
| 15106 | 1 | 1 |  |  |  |  |  |  |  |  | 2 |
| 15110 | 1 |  |  |  |  | 1 |  |  |  |  | 3 |
| 15115 |  |  | 1 |  |  | 1 |  |  |  |  | 2 |
| 15119 |  |  |  |  |  | 1 |  |  |  |  | 1 |
| 15148 |  | 1 | 1 |  |  |  |  |  |  |  | 2 |
| 15172 |  |  | 1 |  |  | 1 |  |  |  |  | 2 |
| 15184 | 1 | 1 |  |  |  |  |  |  |  |  | 2 |
| 15204 | 1 | 2 |  | 1 | 2 | 1 | 2 |  | 1 | 1 | 12 |
| 15212 |  | 1 |  |  |  |  |  |  |  |  | 1 |
| 15218 | 1 |  |  |  |  | 5 | 1 | 1 |  |  | 8 |
| 15223 |  |  |  |  |  | 1 |  |  |  |  | 1 |
| 15235 |  |  |  |  | 1 |  |  | 1 |  |  | 2 |
| 15236 | 1 |  |  |  | 1 |  |  |  |  |  | 2 |
| 15244 |  |  |  |  |  | 1 |  |  |  |  | 1 |
| 15257 | 1 | 1 |  |  |  |  |  |  |  |  | 2 |
| 15259 |  |  |  |  |  | 1 |  |  |  |  | 1 |
| 15261 |  |  |  |  |  |  |  |  |  |  | 1 |
| 15283 | 1 |  |  |  |  |  |  |  |  |  | 1 |
| 15301 | 10 | 11 | 5 | 5 | 3 | 20 | 13 | 5 | 5 | 8 | 85 |
| 15314 |  |  |  |  |  | 2 |  |  |  |  | 2 |
| 15315 |  |  | 1 |  |  |  |  |  |  |  | 1 |
| 15316 |  |  | 2 |  |  | 1 |  |  |  |  | 3 |
| 15326 | 35 | 24 | 12 | 19 | 19 | 67 | 25 | 10 | 9 | 18 | 238 |
| 15328 |  |  |  |  |  | 1 |  |  |  |  | 1 |
| 15340 |  |  |  | 1 |  | 1 |  |  |  |  | 2 |
| 15344 |  | 1 |  |  |  |  | 1 |  |  |  | 2 |
| 15346 |  |  |  |  | 1 |  | 1 |  | 1 |  | 3 |
| 15355 |  |  |  |  |  | 1 |  |  |  |  | 1 |
| 15358 | 1 |  |  |  |  | 1 |  |  |  |  | 1 |
| 15385 |  |  |  |  |  |  |  |  |  |  | 1 |
| 15398 | 1 | 1 |  |  |  |  |  |  |  |  | 2 |
| 15400 |  |  |  |  |  |  | 1 |  |  |  | 1 |
| 15431 |  | 1 |  |  |  | 2 |  |  |  |  | 3 |
| 15440 |  |  | 1 |  |  |  | 1 |  |  | 1 | 3 |
| 15448 |  |  | 1 |  |  |  |  |  |  |  | 1 |
| 15452 | 11 | 3 |  | 1 | 2 | 6 |  |  |  | 1 | 17 |
| 15454 | 1 |  |  |  |  |  |  |  |  |  | 1 |
| 15487 | 2 | 5 |  | 3 | 2 | 4 | 3 | 1 | 1 | 2 | 23 |
| 15497 |  |  |  |  |  | 1 |  |  |  |  | 1 |
| 15508 |  |  |  |  |  | 1 |  |  |  |  | 1 |
| 15519 |  |  |  |  |  | 1 |  |  |  |  | 1 |
| 15535 | 1 |  |  |  | 1 | 2 |  |  |  |  | 4 |
| 15607 | 3 | 2 |  | 1 |  | 3 |  |  |  | 1 | 12 |
| 15613 |  |  |  |  |  |  |  | 1 |  |  | 1 |
| 15625 | 1 |  |  |  |  |  |  |  |  |  | 1 |
| 15628 |  |  |  |  |  |  | 1 |  |  |  | 1 |
| 15661 |  |  |  |  |  | 1 |  |  |  |  | 1 |
| 15662 | 1 |  |  |  |  | 1 |  |  |  |  | 2 |
| 15670 |  |  |  |  | 1 | 1 |  |  |  | 1 | 2 |
| 15693 |  | 1 |  | 2 | 1 | 2 | 3 |  |  | 1 | 11 |
| 15712 |  | 1 |  |  |  |  |  |  |  |  | 1 |
| 15724 | 2 |  |  |  |  |  |  |  |  |  | 2 |
| 15746 |  |  |  |  |  |  |  | 3 |  |  | 3 |
| 15760 |  |  |  |  |  | 1 |  |  |  |  | 1 |
| 15763 |  | 1 |  |  |  | 2 |  |  |  |  | 2 |
| 15777 |  |  |  |  |  | 1 |  |  |  |  | 1 |
| 15784 | 2 | 1 | 1 |  | 1 | 1 |  | 1 |  | 1 | 8 |
| 15789 |  |  |  |  | 1 |  |  |  |  | 1 | 2 |
| 15805 | 1 |  |  |  |  |  |  |  |  |  | 1 |
| 15812 | 1 | 1 |  |  |  |  |  |  |  |  | 2 |
| 15832 | 1 |  |  |  |  |  |  |  |  |  | 1 |
| 15833 |  |  |  | 1 |  |  |  |  |  |  | 1 |
| 15838 |  |  |  |  |  | 2 | 1 |  |  |  | 3 |
| 15840 |  |  |  |  |  |  | 1 |  | 1 |  | 2 |
| 15851 |  |  |  |  |  | 1 |  |  |  |  | 1 |
| 15853 |  |  |  |  |  | 1 |  |  |  | 1 | 2 |
| 15884 | 2 |  | 1 | 2 | 1 | 1 | 1 |  |  |  | 9 |
| 15885 |  |  |  |  |  |  |  | 1 |  |  | 1 |
