# Supplementary material for: Genetic diversities of cytochrome B in Xinjiang Uyghur unveiled its origin and migration history
Source: BMC Genet. 2013 Oct 9;14:100. doi: 10.1186/1471-2156-14-100 (PMC3852047; doi:10.1186/1471-2156-14-100)
Supplement: Additional file 2: Table S2 — Frequency of haplotype of MTCYB. [file 1471-2156-14-100-S2.doc]

**Supplemental Table 2**  **Frequency of haplotype of MTCYB**

| Haplotype | Polymorphic Positions | A  KK | AT | BO | GU | HO | KA | KO | KU | SA | TU | Total |
| --- | --- | --- | --- | --- | --- | --- | --- | --- | --- | --- | --- | --- |
|  |  | 35 | 24 | 13 | 19 | 19 | 67 | 25 | 10 | 10 | 18 | 240 |
| 1 |  |  |  |  |  |  | 1 |  |  |  |  | 1 |
| 2 | 14766T,14905A,15326G,15452A,15607G | 2 | 1 |  | 1 |  | 2 |  |  |  |  | 6 |
| 3 | 14766T,14794A,14905A,15326G,15452A,15607G | 1 |  |  |  |  |  |  |  |  |  | 1 |
| 4 | 14766T,15257A,15326G,15452A,15662G,15805G,15812A | 1 |  |  |  |  |  |  |  |  |  | 1 |
| 5 | 14766T,14783C,15043A,15106A,15184C,15301A,15326G | 1 | 1 |  |  |  |  |  |  |  |  | 2 |
| 6 | 14766T,14783C,15043A,15301A,15326G,15724G | 2 |  |  |  |  |  |  |  |  |  | 2 |
| 7 | 14766T, 14783C,15043A,15204C,15301A,15326G,15487T | 1 | 2 |  | 1 | 2 | 1 | 2 |  | 1 | 1 | 11 |
| 8 | 14766T,14783C,15043A,15110A,15301A,15326G | 1 |  |  |  |  | 1 |  | 1 |  |  | 3 |
| 9 | 14766T,15067C,15326G,15625T | 1 |  |  |  |  |  |  |  |  |  | 1 |
| 10 | 14766T,14783C,15043A,15301A,15326G,15784C | 1 |  | 1 |  |  |  |  |  |  |  | 2 |
| 11 | 14766T,14783C,15043A,15301A,15326G | 2 | 3 | 1 | 1 | 1 | 5 | 8 |  | 3 | 3 | 27 |
| 12 | 14766T,15043A,15326G | 1 |  |  |  |  |  |  |  |  |  | 1 |
| 13 | 14766T,15326G | 3 | 4 |  | 3 | 1 | 8 | 4 | 1 | 2 | 5 | 31 |
| 14 | 14766T,15326G,15784C,15884C | 1 |  |  |  |  |  |  |  |  |  | 1 |
| 15 | 15326G | 7 | 3 | 2 | 3 | 3 | 10 | 2 | 1 | 1 | 1 | 33 |
| 16 | 14766T,15326G,15693C | 1 | 1 |  | 2 |  | 1 | 2 |  |  |  | 7 |
| 17 | 14766T,15326G,15535T | 1 |  |  |  | 1 | 2 |  |  |  |  | 4 |
| 18 | 14766T,14783C,15043A,15204C,15236G,15301A,15326G,15487T | 1 |  |  |  |  |  |  |  |  |  | 1 |
| 19 | 14766T,15326G,15884C | 1 |  | 2 | 1 | 1 | 1 | 1 |  |  |  | 7 |
| 20 | 14766T,15049T,15283C,15326G | 1 |  |  |  |  |  |  |  |  |  | 1 |
| 21 | 14766T,15326G,15454C | 1 |  |  |  |  |  |  |  |  |  | 1 |
| 22 | 14766T,14783C,15043A,15301A,15326G,15832T | 1 |  |  |  |  |  |  |  |  |  | 1 |
| 23 | 14766T,15326G,15358G | 1 |  |  |  |  |  |  |  |  |  | 1 |
| 24 | 14766T,15326G,15398G | 1 | 1 |  |  |  |  |  |  |  |  | 2 |
| 25 | 14798C,15218G,15326G | 1 |  |  |  |  |  |  |  |  |  | 1 |
| 26 | 14766T,15148A,15326G |  | 1 |  |  |  |  |  |  |  |  | 1 |
| 27 | 14766T,14783C,15043A,15301A,15326G,15431A |  | 1 |  |  |  | 1 |  |  |  |  | 2 |
| 28 | 14766T,15212G,15257A,15326G,15452A,15812A |  | 1 |  |  |  |  |  |  |  |  | 1 |
| 29 | 14766T,14783C,14978G,15043A,15301A,15326G,15344C,15487T |  | 1 |  |  |  |  | 1 |  |  |  | 2 |
| 30 | 14753T,14766T,14905A,15326G,15452A,15607G,15763G |  | 1 |  |  |  |  |  |  |  |  | 1 |
| 31 | 14766T,14783C,15043A,15301A,15326G,15487T |  | 1 |  | 1 |  | 1 |  |  |  |  | 3 |
| 32 | 14766T,14783C,15043A,15301A,15326G,15712G |  | 1 |  |  |  |  |  |  |  |  | 1 |
| 33 | 14766T,14783C,15043A,15301A,15326G,15487T,15784C |  | 1 |  |  |  |  |  | 1 |  |  | 2 |
| 34 | 14751T,14766T,14783C,15043A,15301A,15315T,15326G |  |  | 1 |  |  |  |  |  |  |  | 1 |
| 35 | 14766T,14783C,14815T,14927G,15043A,15301A,15326G,15440C,15448A |  |  | 1 |  |  |  |  |  |  |  | 1 |
| 36 | 14766T,15148A,15172A,15326G |  |  | 1 |  |  |  |  |  |  |  | 1 |
| 37 | 14766T,14793G,15316G,15326G |  |  | 1 |  |  |  |  |  |  |  | 1 |
| 38 | 15115C,15326G |  |  | 1 |  |  | 1 |  |  |  |  | 2 |
| 39 | 14766T,15316G,15326G |  |  | 1 |  |  | 1 |  |  |  |  | 2 |
| 40 | 14766T,14783C,14927G,15043A,15301A,15440C |  |  | 1 |  |  |  |  |  |  |  | 1 |
| 41 | 14763C,14766T,14783C,14978G,15043A,15301A,15326G,15487T |  |  |  | 1 |  |  |  |  |  |  | 1 |
| 42 | 14766T,14783C,14979C,15043A,15301A,15326G |  |  |  | 1 |  | 2 |  |  |  |  | 3 |
| 43 | 14766T,14831A,15031T,15326G |  |  |  | 1 |  |  |  |  |  |  | 1 |
| 44 | 14766T,15326G,15884A |  |  |  | 1 |  |  |  |  |  |  | 1 |
| 45 | 15326G,15833T |  |  |  | 1 |  |  |  |  |  |  | 1 |
| 46 | 15326G,15340G |  |  |  | 1 |  | 1 |  |  |  |  | 2 |
| 47 | 14766T,14935C,15043A,15061G,15236G,15326G |  |  |  |  | 1 |  |  |  |  |  | 1 |
| 48 | 14872T,15326G |  |  |  |  | 1 | 1 |  |  |  |  | 2 |
| 49 | 14751T,14766T,15326G |  |  |  |  | 1 |  |  |  |  | 1 | 2 |
| 50 | 14766T,15326G,15346A |  |  |  |  | 1 |  | 1 |  | 1 |  | 3 |
| 51 | 14766T,15326G,15670C |  |  |  |  | 1 |  |  |  |  |  | 1 |
| 52 | 14766T,14798C,15326G,15452A |  |  |  |  | 2 |  |  |  |  |  | 2 |
| 53 | 14766T,15235G,15326G,15784C |  |  |  |  | 1 |  |  |  |  |  | 1 |
| 54 | 14766T,15067C,15326G |  |  |  |  | 1 |  |  |  |  |  | 1 |
| 55 | 14766T,15326G,15693C,15789T |  |  |  |  | 1 |  |  |  |  | 1 | 2 |
| 56 | 14766T,14798C,15326G,15760T |  |  |  |  |  | 1 |  |  |  |  | 1 |
| 57 | 14766T,15119A,15326G,15355A,15693C |  |  |  |  |  | 1 |  |  |  |  | 1 |
| 58 | 14766T,15326G,15452A |  |  |  |  |  | 1 |  |  |  |  | 1 |
| 59 | 14766T,14783C,15043A,15301A,15326G,15773A |  |  |  |  |  | 2 |  |  |  |  | 2 |
| 60 | 14766T, 14783C,15043A, 15301A,15326G,15777C |  |  |  |  |  | 1 |  |  |  |  | 1 |
| 61 | 14766T, 14783C,15043A, 15301A,15326G,15519C |  |  |  |  |  | 1 |  |  |  |  | 1 |
| 62 | 14756G,14766T,15218G,15326G |  |  |  |  |  | 1 |  |  |  |  | 1 |
| 63 | 14766T, 14783C,15218G 15043A, 15301A,15326G,15487T,15784C |  |  |  |  |  | 1 |  |  |  |  | 1 |
| 64 | 14766T, 14783C,15043A, 15301A,15326G,15838A |  |  |  |  |  | 1 |  |  |  |  | 1 |
| 65 | 14766T,15326G,15497A |  |  |  |  |  | 1 |  |  |  |  | 1 |
| 66 | 14766T,15314A,15326G,15452A |  |  |  |  |  | 2 |  |  |  |  | 2 |
| 67 | 14766T,14905A,15326G,15452A,15607G,15838A,15853T |  |  |  |  |  | 1 |  |  |  |  | 1 |
| 68 | 14766T, 14783C,15043A, 15259T,5301A,15326G,15413A |  |  |  |  |  | 1 |  |  |  |  | 1 |
| 69 | 14753T,14766T,14767C,15326G |  |  |  |  |  | 1 |  |  |  |  | 1 |
| 70 | 14766T,14793G,15218G,15326G |  |  |  |  |  | 2 | 1 |  |  |  | 3 |
| 71 | 14766T,15326G,15385T |  |  |  |  |  | 1 |  |  |  |  | 1 |
| 72 | 14753T,15172A,15326G |  |  |  |  |  | 1 |  |  |  |  | 1 |
| 73 | 14766T, 14783C,15043A,15244G,15301A,15326G,15487T |  |  |  |  |  | 1 |  |  |  |  | 1 |
| 74 | 14766T,14831A,15055C,15223T,15326G,15508T,15662G,15851G |  |  |  |  |  | 1 |  |  |  |  | 1 |
| 75 | 14750G,14766T,14793G,15218G,15326G |  |  |  |  |  | 1 |  |  |  |  | 1 |
| 76 | 14766T,15326G,15661T |  |  |  |  |  | 1 |  |  |  |  | 1 |
| 77 | 14766T,14783C,15043A,15301A,15326G,15328G |  |  |  |  |  | 1 |  |  |  |  | 1 |
| 78 | 14766T,14783C,14905A15043A,15301A,15326G, |  |  |  |  |  | 1 |  |  |  |  | 1 |
| 79 | 14766T,15218G,15326G |  |  |  |  |  | 1 |  |  |  |  | 1 |
| 80 | 14766T,14783C,14801C,14804T,15043A,15301A,15326G,15440C,15838A,15840C |  |  |  |  |  |  | 1 |  |  |  | 1 |
| 81 | 14766T,15326G,15628T,15693C |  |  |  |  |  |  | 1 |  |  |  | 1 |
| 82 | 14766T,14783C,15043A,15301A,15326G,15400T |  |  |  |  |  |  | 1 |  |  |  | 1 |
| 83 | 14766T,14783C,15043A,15301A,15326G,15746G |  |  |  |  |  |  |  | 3 |  |  | 3 |
| 84 | 14766T,14793G,14893G,14971C,15218G,15326G |  |  |  |  |  |  |  | 1 |  |  | 1 |
| 85 | 14766T,15326G,15613G |  |  |  |  |  |  |  | 1 |  |  | 1 |
| 86 | 14766T,15043A,15235G,15326G,15885T |  |  |  |  |  |  |  | 1 |  |  | 1 |
| 87 | 14766T |  |  |  |  |  |  |  |  | 1 |  | 1 |
| 88 | 14766T,14783C,15022T,15043A,15301A,15326G,15840G |  |  |  |  |  |  |  |  | 1 |  | 1 |
| 89 | 14766T,14783C,14857C,15043A,15301A,15326G |  |  |  |  |  |  |  |  |  | 2 | 2 |
| 90 | 14766T,14783C,15043A,15301A,15326G,15670C |  |  |  |  |  |  |  |  |  | 1 | 1 |
| 91 | 14766T,14783C,15043A,15261A,15301A,15326G,15487T,15784C |  |  |  |  |  |  |  |  |  | 1 | 1 |
| 92 | 14766T,14783C,14815T,14927G,15043A,15301A,15326G,15440C |  |  |  |  |  |  |  |  |  | 1 | 1 |
| 93 | 14766T,15326G,15452A,15607G,15853T |  |  |  |  |  |  |  |  |  | 1 | 1 |

AK :Aksu; AT : Atush ; BO :Bortala ; GU : Gulja; HO : Hotan; KA : Kashgar; KO : Korla ; KU : Kumul; SA : Sanji; TU: Turpan.
